# The N-terminus of the *Clostridioides difficile* transferase A component directs toxin activity and potency

**DOI:** 10.1128/mbio.02405-24

**Published:** 2024-11-29

**Authors:** Robin M. Mullard, Michael J. Sheedlo

**Affiliations:** 1 Department of Pharmacology, University of Minnesota Medical School, Minneapolis, Minnesota, USA; St. Jude Children's Research Hospital, Memphis, Tennessee, USA

**Keywords:** *Clostridioides difficile*, toxins, pore-forming toxins, mechanisms of action

## Abstract

*Clostridioides difficile* infection is the leading cause of antibiotic-associated, hospital-acquired diarrhea in the USA; the pathology of which is mediated by toxins. The presence of a toxin known as the *C. difficile* Transferase (CDT) in some clinical isolates is linked to severe symptoms including increased incidence of reinfection and higher rates of mortality. Despite its apparent importance to *C. difficile* pathology, a mechanistic model of how CDT intoxicates cells remains incomplete. Here, we describe a motif composed of acidic and basic residues (the KDKEK motif) that is essential for toxin function. Using Cryogenic Electron Microscopy (Cryo-EM), we highlight an orientation of the KDKEK motif wherein the acidic residues engage structures thought to play an important role during toxin delivery. We thus present a model wherein these interactions prime CDT for entry into host cells. We expect that this model can be extrapolated to other bacterial toxins to understand how they enter cells.

**IMPORTANCE:**

*Clostridioides difficile* is the leading cause of hospital-acquired infectious diarrhea in the USA. The pathology that accompanies infection is triggered by toxins produced by the bacterium. One of these, the *C. difficile* Transferase (CDT), has been associated with poorer patient outcomes, although a direct connection to CDT activity has remained elusive. Herein, we present new insight into the mechanism of CDT intoxication and define two regions of the toxin as important for its activity. Moreover, we have generated mutants of CDT that retain the ability to assemble but can no longer intoxicate host cells. In the future, we expect these mutants will serve as valuable tools to help elucidate the role of CDT during infection.

## INTRODUCTION

*Clostridioides difficile* is a gram-positive pathogen and one of the leading causes of antibiotic-associated, hospital-acquired diarrhea in the USA (1). The pathology associated with *C. difficile* infection is mediated by the activity of up to three toxins that are secreted by the bacterium at the site of infection (2, 3). One of these toxins, known as the *C. difficile* Transferase (CDT), is associated with clinical strains of *C. difficile* (including the so-called “hypervirulent” strain) that have been linked to severe symptoms, increased incidence of reinfection, and high rates of mortality (4–6). Despite its apparent importance, an incomplete model of CDT intoxication persists.

CDT is a member of the Iota family of binary toxins, a group of homologous proteins that also includes the *Clostridium perfringens* Iota and *Clostridium spiroforme* Transferase (CST) toxins (7–9). All three toxins are assembled from two polypeptide chains: an enzymatically active “A” component (CDTa, Iota-a, and CSTa) and a cell-binding and delivery “B” component (CDTb, Iota-b, and CSTb) (3, 7, 10). A mechanism describing CDT intoxication has been assembled using data generated from all three family members, due to their high degree of homology. This mechanism starts with the localization of CDTb to host cells through interactions with a receptor known as Angulin-1 (or LSR) (11). On the cell surface, the A and B components assemble into a heterooligomeric structure (CDT, also known as the holotoxin), and the complex enters the host cell through endocytosis. Once inside the endosome, the establishment of a potential and pH gradient is thought to trigger the process of CDTa translocation during which CDTa unfolds, passes through a membrane-spanning channel formed by CDTb, and enters the host cytoplasm (12, 13). Due to the complexity of the proposed mechanism, biochemical studies have predominantly focused on understanding the functionality of each individual component.

The pro-peptide form of CDTa is composed of 463 residues with an apparent secretion signal located at the N-terminus that is expected to be removed upon secretion from the bacterium (3). Predictions place the secretion signal cleavage site of CDTa between residues 34 and 42, although a specific site has not yet been experimentally defined (3, 14). The mature form of CDTa consists of at least three elements: (i) an N-terminal unstructured region (residues 35–62), (ii) a non-catalytic pseudo-ADP-ribosyltransferase domain (pADPRT, residues 63–256), and (iii) a catalytically active ADP-ribosyltransferase domain (ADPRT, residues 271–463) (15). Despite its high conservation within the family of Iota toxins, no definitive role for the N-terminal unstructured region has been defined. Conversely, both the pADPRT and the ADPRT are needed to evoke the cell rounding phenotype associated with intoxication (16). Although the pADPRT is presumed to possess no enzymatic activity, it acts as a scaffold during holotoxin assembly (17, 18). Interestingly, portions of a five-helix subdomain within the N-terminus of the pADPRT, referred to as the “adaptor,” are not needed for toxin assembly but are required for cytotoxicity, indicating that motifs within the adaptor may drive toxin delivery (18). Unlike the pADPRT, the ADPRT is enzymatically active and functions by modifying host cell actin and the actin-related protein 2/3 with ADP-ribose to induce cytotoxicity (19, 20). The activity of CDTa results in the collapse of the host cell cytoskeleton, resulting in a distinctive cell rounding phenotype *in vitro*, which has become the hallmark of CDT-induced cytotoxicity (21).

**Fig 1:**
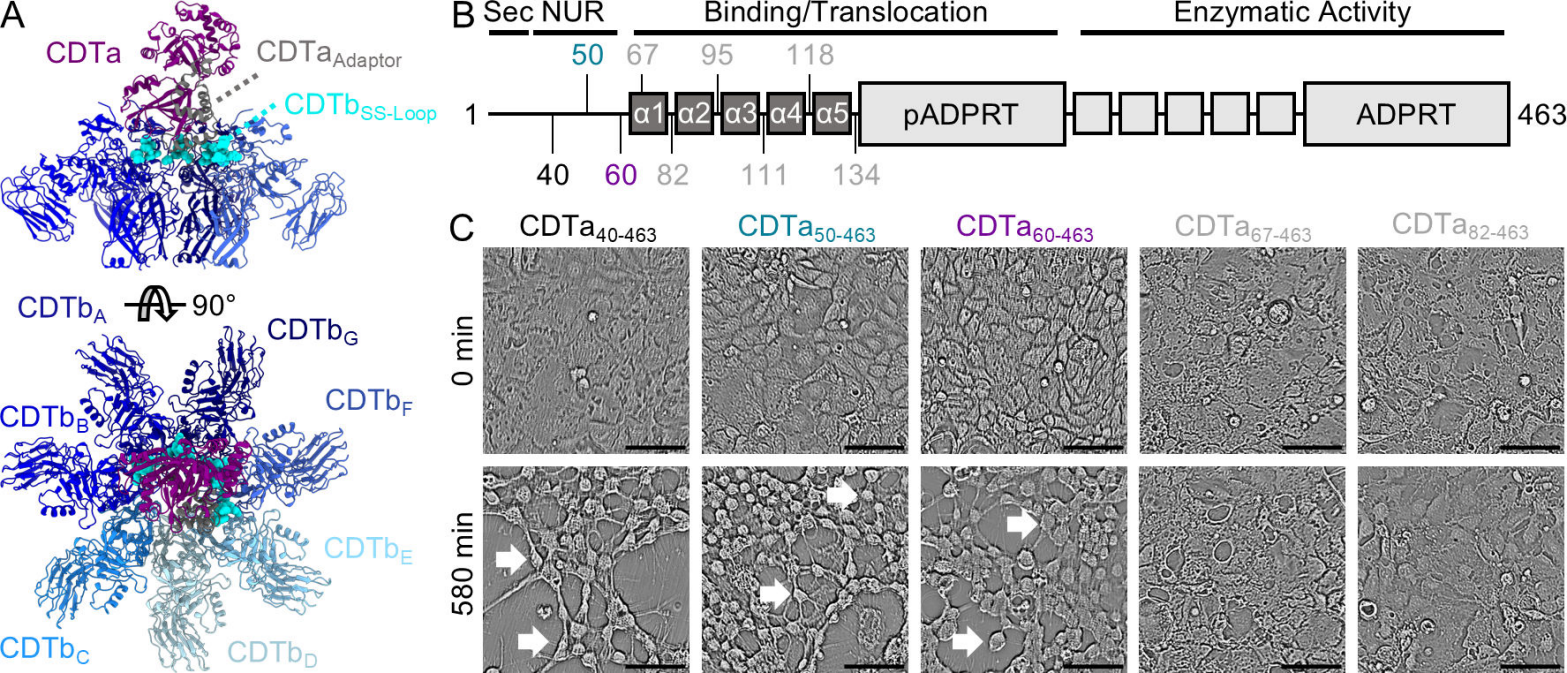
Cytotoxic effects of CDTa truncations. (**A**) A schematic representation of the structure of CDT previously reported (PDB 7YVQ). CDTa is shown in purple, and CDTb in various shades of blue. The CDTb SS-Loop is shown in cyan, and the CDTa adaptor in gray. (**B**) Nine different CDTa constructs, beginning at the indicated positions, were generated to assess the role of the adaptor and NUR during intoxication. NUR, N-terminal unstructured region; Sec, secretion signal; pADPRT, pseudo-ADP-ribosyltransferase domain; ADPRT, ADP-ribosyltransferase domain. (**C**) The activity of the indicated constructs on Caco-2 cells in the presence of CDTb. Scale bar indicates 100 µm. White arrows point to cells that have been intoxicated.

In contrast to CDTa, CDTb is structurally complex and composed of five domains termed D1, D2, D3, D3’, and D4, which are involved in regulation (D1), pore formation (D2), oligomerization (D2 and D3), and host cell interactions (D3’ and D4 are also known as RBD1 and RBD2) (22, 23). In the context of intoxication, CDTb acts as the delivery apparatus, forming a membrane spanning pore within the membrane of host cells to facilitate the delivery of CDTa into the cytoplasm (22). The functional toxin moiety assembles from one molecule of CDTa and one CDTb heptamer with the majority of interactions occurring between the pADPRT domain of CDTa and the D2 domain of CDTb (17, 18). Notably, four of the seven CDTb protomers contact CDTa through the same structure; a loop known as the “SS-Loop” (also referred to as the NSS-Loop in CDTb and NSQ-Loop in Iota-b, Fig. 1A). The sheer number of interactions between the SS-Loop and CDTa suggests that it plays a pivotal role during intoxication. Indeed, a recent study has described flexibility within the SS-Loop of CDTb wherein two different conformations, described as Loop-in and Loop-out, were defined. These conformations were observed in concert with local unfolding of the CDTa N-terminus, and it was thus suggested that the dynamics of the SS-Loop play an important role during CDTa translocation (17). In the holotoxin, CDTa is oriented in the center of the CDTb heptamer (17, 18, 24). This arrangement places the N-terminus of CDTa near an essential feature of the CDTb delivery apparatus known as the Φ-clamp, a structure that is conserved among binary toxins and thought to act as a gate to regulate toxin delivery (22, 25). Little is known about how CDTa moves through the CDTb pore, and much of our understanding of this process has relied on studies conducted on the prototypical anthrax toxin of *Bacillus anthracis* as the delivery components of both systems are structurally related (26, 27). However, differences in the electrostatic character of the delivery components and the radically different structures of their corresponding enzymatic cargo suggest the possibility of divergent translocation mechanisms among the two toxins.

In this study, we explore the role of features within the N-terminus of CDTa during intoxication. Through our work, we have uncovered a motif (the KDKEK motif) within the CDTa N-terminus that is absolutely required for intoxication and present structural evidence for its engagement with the CDTb SS-Loop. We propose that the KDKEK motif plays a role in orienting features within the N-terminal unstructured region of CDTa near adjacent SS-Loops to effectively prime CDTa for delivery. In support of this model, we show that constructs containing the N-terminal unstructured region exhibit potencies that are ~500-fold greater than constructs that do not contain this feature. Furthermore, we demonstrate that mutating residues within this segment severely impacts the potency of the toxin. Finally, we propose a model for the priming of CDTa for delivery into host cells and predict that portions of the model may be extrapolated even to distantly related toxins (such as anthrax toxin) due to the presence of analogous structural features. Thus, we expect that these data will serve as a general guide for understanding how binary toxins access their host.

## RESULTS

### Residues 60–66 of CDTa are required for intoxication

To broadly assess the role of the CDTa N-terminus during intoxication, we first generated a panel of CDTa constructs truncated at various points throughout the N-terminal unstructured region and the adaptor subdomain of the pADPRT. Three of these constructs (CDTa_40-463_, CDTa_50-463_, and CDTa_60-463_) were designed to start at positions within the N-terminal unstructured region, and the remaining six (CDTa_67-463_, CDTa_82-463_, CDTa_95-463_, CDTa_111-463_, CDTa_118-463_, and CDTa_134-463_) begin in the loops situated between each of the five helices of the adaptor (Fig. 1B). All nine constructs were purified, mixed with CDTb, and applied to Caco-2 cells in tissue culture to assess their ability to intoxicate cells (Fig. S1A). Of these nine constructs, only those containing residues 60–66 (CDTa_40-463_, CDTa_50-463_, and CDTa_60-463_) were found to induce cell rounding over a 580-minute incubation period, indicating the entire adaptor subdomain is required for activity. Interestingly, the N-terminal unstructured region was not required, in its entirety, to induce the cytotoxic effects associated with CDT administration (Fig. 1C; Fig. S1B). To confirm that the observed inactivity was not due to defects in holotoxin formation, we used biolayer interferometry to assess the affinity and rate of association between CDTa and oligomeric CDTb. From our analysis, we derived comparable dissociation constants (K_d_) for CDTa_60-463_ (9.5 nM) and CDTa_67-463_ (18.5 nM). Similarly, the derived rates of holotoxin maturation (k_ON_) for each construct were in close agreement (CDTa_60-463_: 8 × 10^5^ M^−1^ sec^−1^ and CDTa_67-463_: 4 × 10^5^ M^−1^ sec^−1^, Fig. S2). Next, we assessed the ability of each CDTa construct used in this study to ADP-ribosylate host cell proteins using an immunoblot-based strategy. From this analysis, we confirmed that CDTa_40-463_, CDTa_50-463_, CDTa_60-463_, and CDTa_67-463_ were active in host cell lysates (Fig. S1C). Because CDTa_67-463_ cannot induce cytopathic cell rounding despite being capable of effectively modifying actin within the host cell, we suggest that residues 60–66 of CDTa play an important role that is downstream of toxin assembly but upstream of host cell protein modification.

### The CDTb SS-Loop engages the CDTa N-terminal unstructured region in the assembled toxin

**Fig 2:**
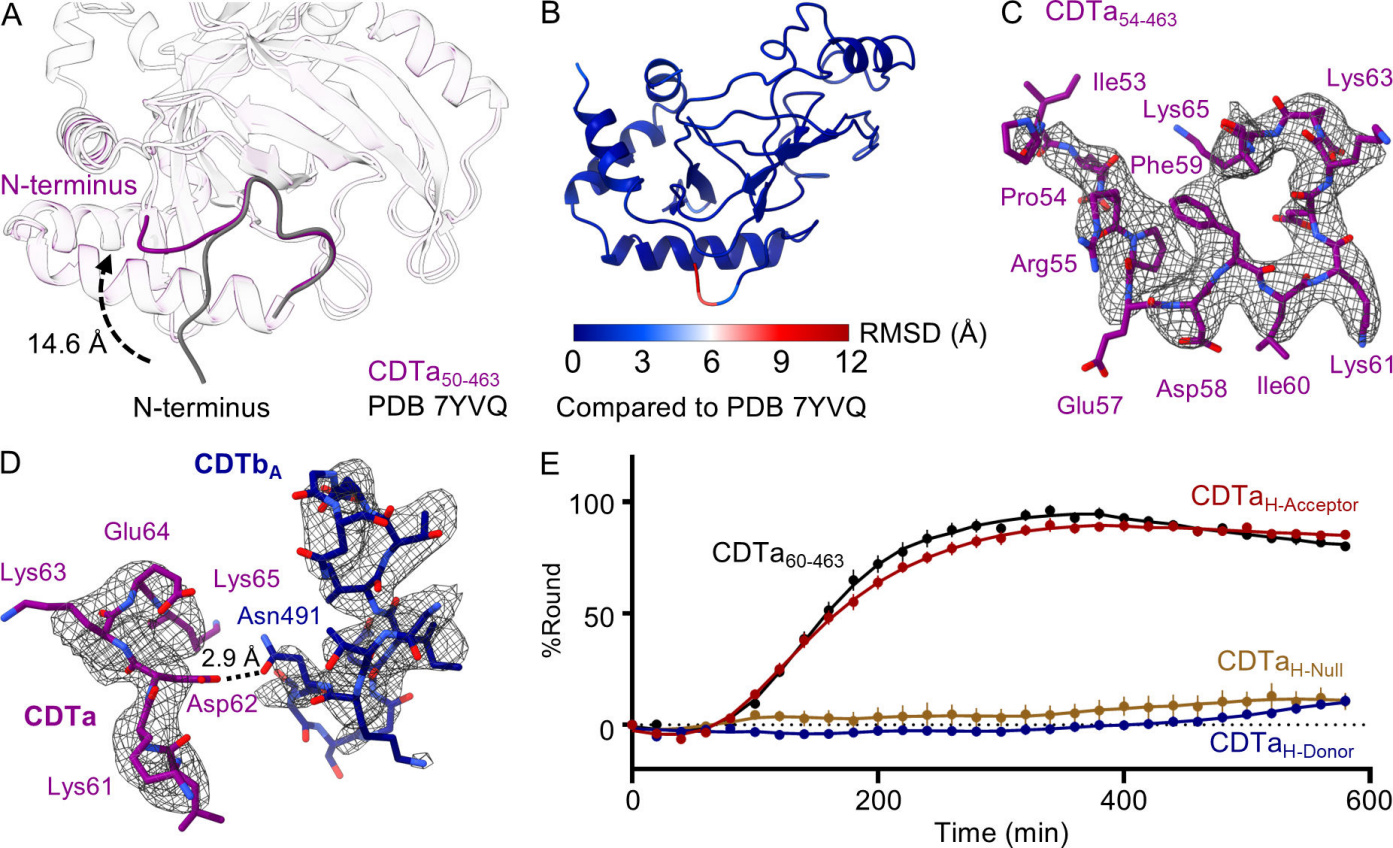
The N-terminal unstructured region is situated near the CDTb SS-Loop in the assembled toxin. (**A**) The N-terminal unstructured region of CDTa adopts a unique conformation in the structure presented here, shifted 14.6 Å to a lateral position situated near the CDTb SS-Loop. (**B**) The model of the CDTa pADPRT domain extracted from the structure described here. A comparison of the CDTa structure with a previously determined structure (PDB 7YVQ) illustrating differences between the two as the RMSD of backbone atoms. Note, only the N-terminus deviates from the previously determined structure. (**C**) Density corresponding to residues 53–65 of CDTa. (**D**) The SS-Loop of CDTb_A_ is shown in navy and is situated near the KDKEK motif of CDTa (purple). The KDKEK motif of CDTa is positioned such that Asp62 is oriented to contact the Asn491 from the SS-Loop of CDTb_A_ (inset). (**C**) Results of a cell rounding assay conducted using CDTa_H-Null_ (yellow), CDTa_H-Donor_ (blue), and CDTa_H-Acceptor_ (red). Results are compared to CDTa_60-463_ (black). Error bars represent the standard error of measurement.

Because residues 60–66 of CDTa do not contribute to holotoxin assembly, we hypothesized this segment primes the N-terminus of CDTa for translocation. To probe this possibility, we determined the structure of CDTa_50-463_ in complex with a CDTb heptamer by Cryogenic Electron Microscopy (Cryo-EM) to a global resolution of 3.6 Å (Fig. S3A through D; Table S1). In the map reported here, the ADPRT of CDTa is apparently flexible, limiting the resolution and interpretation of this region of the map. Consequently, model construction was restricted to the CDTa N-terminal unstructured region and pADPRT (residues 52–104 and 119–260, Fig. S4A). The position and orientation of CDTa are consistent with previously reported structures wherein the N-terminus of CDTa adopts a “folded” conformation (global root mean square deviation of 1.3 Å compared to PDB 7YVQ), supporting the current model of assembly (Fig. S4B) (28). However, we note a significant change in the orientation of the N-terminal unstructured region of CDTa wherein residues 53–59 are situated perpendicular to the CDTb pore in contrast to the vertical orientation previously described (Fig. 2A through C) (17). In response, the SS-Loops of several CDTb protomers reorganize to adopt unique conformations that result in apparent contacts with residues 53–62 of CDTa by the SS-Loops of Chains A and G of the CDTb heptamer (CDTb_A_ and CDTb_G_, Fig. S4B). This places the critical segment formed by residues 60–66 of CDTa between the SS-Loops of CDTb_A_ and CDTb_B_ (Fig. 2D). Based on this position, we predicted that the SS-Loop may interact with residues within the CDTa N-terminus through hydrogen bonds facilitating toxin activity. In support of this idea, this region contains a highly conserved motif composed of alternating acidic and basic residues (which we have termed the KDKEK motif) that may act as hydrogen bond acceptors and donors, respectively (Fig. S5A). We also note that, although the resolution of this map prohibits the direct assignment of hydrogen bonds, Asn491 of CDTb is correctly positioned to engage Asp62 of the KDKEK motif (Fig. 2D). To test this possibility, we generated a small panel of mutants with the goal of evaluating the effect that hydrogen bond donors and acceptors have on toxin activity. We generated three constructs wherein the KDKEK motif was mutated such that it contained no potential for hydrogen bond formation through side chain interactions (CDTa_H-Null_), contained only hydrogen bond donors (CDTa_H-Donor_), and contained only hydrogen bond acceptors (CDTa_H-Acceptor_) (Fig. S5B and C). To rule out the possibility that these mutations result in gross structural changes, all three constructs were subjected to analysis by biolayer interferometry and were shown to interact with oligomeric CDTb with affinities comparable to wild-type CDTa (K_d_ of 13.4, 4.3, and 6.8 nM, for CDTa_H-Null_, CDTa_H-Donor_, and CDTa_H-Acceptor_, respectively, Fig. S5E). Similarly, all three constructs retain activity in host cell lysates, albeit at differential levels (Fig. S1C). Of the three constructs, only CDTa_H-Acceptor_ was found to induce cytotoxicity in Caco-2 cells over the course of a 580-minute incubation period (Fig. 2C). These results indicate that acidic residues within the KDKEK motif (Asp62 and Glu64) play a key role in facilitating CDT activity, potentially through critical interactions with the CDTb SS-Loop. In support of this assertion we have shown that constructs containing only Asp62 or Glu64 retain the ability to round Caco-2 cells in tissue culture (Fig. S1D).

### The N-terminal unstructured region enhances toxin activity and potency

**Fig 3:**
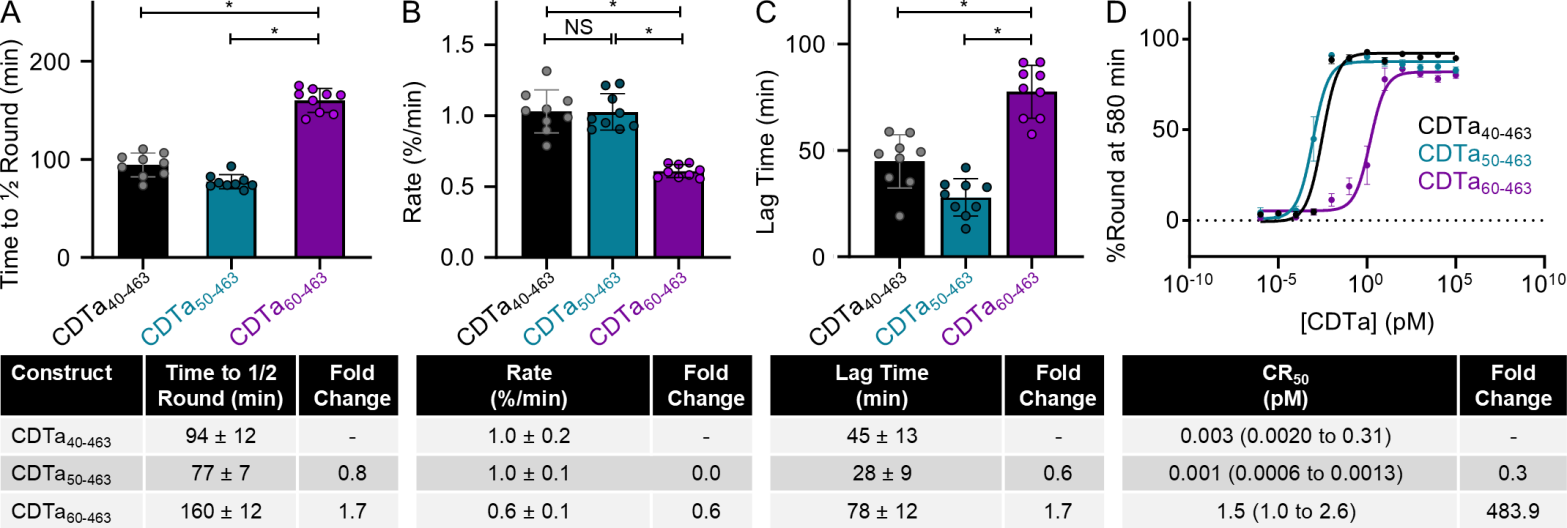
Assessment of the activity of CDTa truncations and mutants. (**A**) The time taken for each construct to round half of all cells in the field of view at a final concentration of 1 pM (top). Constructs were compared with a one-way ANOVA; an asterisk indicates a *P* value < 0.0001. Error bars represent standard deviation. The calculated average rates for each construct are reported in the table below. The change in rate is compared with CDTa_40-463_. (**B**) The rounding rate of each construct at 1 pM (top). Constructs were compared with a one-way ANOVA; an asterisk indicates a *P* value < 0.0001, NS indicates no statistically significant difference. Error bars represent standard deviation. The average rate for each construct is reported in the table below. The apparent change in rate is compared with CDTa_40-463_. (**C**) The time it takes for cells to begin rounding for each construct (top). The activities were compared with a one-way ANOVA; an asterisk indicates a *P* value < 0.0001. Error bars represent standard deviation. Average lag times are included in the table below, and the rates are compared with CDTa_40-463_. (**D**) A titration to determine the activity of all five constructs. The calculated CR_50_ values are listed in the table below. The change in CR_50_, as compared with CDTa_40-463_ value, is listed on the right. Error bars represent standard deviation.

Because the KDKEK motif of CDTa plays a critical role in mediating the activity of CDT during intoxication, we expected that all constructs containing this motif would exhibit similar levels of activity. To test this, we evaluated the rates and efficiencies of the three active CDTa constructs (CDTa_40-463_, CDTa_50-463_, and CDTa_60-463_). Under this experimental setup, we tracked the activity of each construct, monitoring the evolution of cell rounding every 20 minutes for 5 hours. At a concentration of 100 pM, the activities of CDTa_40-463_ and CDTa_50-463_ were virtually indistinguishable, taking an average of 94 ± 12 minutes and 77 ± 7 minutes to round half of all cells in the field of view, respectively. In contrast, CDTa_60-463_ took significantly longer, averaging 160 ± 12 minutes to round half of all cells in the field of view (Fig. 3A; Fig. S6A). Notably, the rate at which cell rounding occurred was modestly impaired for CDTa_60-463_ (0.6 ± 0.1%/min) when compared to CDTa_40-463_ (1.0 ± 0.2%/min) and CDTa_50-463_ (1.0 ± 0.1%/min) (Fig. 3B). Similarly, CDTa_60-463_ took significantly longer (78 ± 12 minutes) to start rounding cells than CDTa_40-463_ (45 ± 13 minutes) and CDTa_50-463_ (28 ± 9 minutes), resulting in a lag in the evolution of the intoxication phenotype (Fig. 3C; Fig. S6B). Notably, the observed disparities in activity cannot be overcome by increasing the concentration of CDTa, as CDTa_60-463_ remained defective across all three parameters tested even at a final concentration of 100 nM (Fig. S7C). We next assessed the effect that the length of the N-terminal unstructured region has on toxin potency. To that end, CDTa_40-463_, CDTa_50-463_, and CDTa_60-463_ were titrated on Caco-2 cells in the presence of CDTb, ranging in concentration from 100 nM to 1 aM. The efficiency of each construct (CR_50_) was then defined as the concentration at which half of all cells are round after a 580-minute incubation period in accordance with assays conducted on similar toxins (29). Strikingly, CDTa_40-463_ and CDTa_50-463_ exhibited potencies far greater than that of CDTa_60-463_ (CR_50_ of 0.003 pM for CDTa_40-463_, 0.001 pM for CDTa_50-463_, and 1.5 pM for CDTa_60-463_, Fig. 3D; Fig. S8). Given the magnitude of the decrease in potency (~500-fold), we sought to confirm that the truncation of the N-terminal unstructured region did not affect the dynamics of holotoxin formation, deriving similar affinities (K_d_, 9.0 nM for CDTa_40-463_ and 5.7 nM for CDTa_50-463_) and rates of association (k_ON_, 12 × 10^5^ M^−1^ s^−1^ for CDTa_40-463_ and 14 × 10^5^ M^−1^ s^−1^ for CDTa_50-463_) for each construct (Fig. S9). Together, these results highlight a previously unappreciated role for residues 50–59 within the CDTa N-terminal unstructured region during intoxication.

To assess how the identity of residues within the CDTa N-terminal unstructured region contributes to the enhanced potency we observed, we constructed mutants of CDTa wherein the N-terminus of CDTa_40-463_ and CDTa_50-463_ were replaced with penta-alanine blocks (CDTa_5Ala45-463_ and CDTa_5Ala55-463_, Fig. S10A). In cytotoxic cell rounding assays, we derived a similar potency for CDTa_5Ala45-463_ (CR_50_ of 0.003 pM) as the wild-type construct, indicating that residues 40–44 do not contribute to the enhanced potency observed for these constructs. Conversely, the potency of CDTa_5Ala55-463_ was much lower (CR_50_ of 35.2 pM) than the corresponding wild-type construct, suggesting residues 50–54 direct the increase in potency of constructs containing residues 50–59 (Fig. S10C and D).

## DISCUSSION

**Fig 4:**
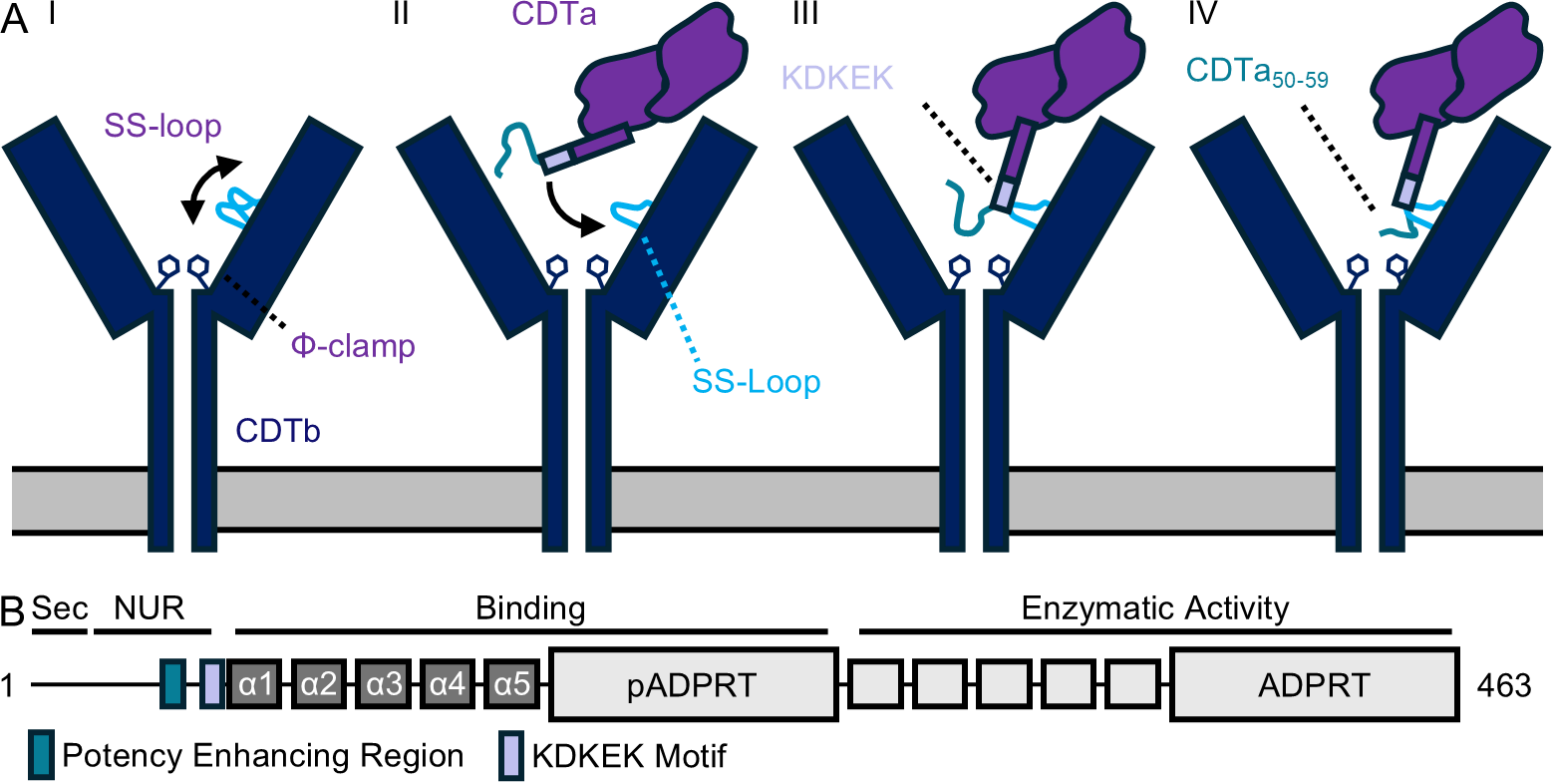
Features of CDTa involved in toxin priming. (**A**) The CDTb (blue) SS-Loop (light blue) has been shown to sample different conformations (I). Upon CDTa (purple) binding, the adaptor subdomain shifts to contact the SS-Loop (II). Interactions between the KDKEK motif (lavender) of the CDTa adaptor and the SS-Loop restrict motion within the CDTa N-terminus (III). With restricted flexibility, residues 50–59 of the CDTa unstructured region (cyan) are repositioned to contact the SS-Loop of adjacent CDTb protomers (IV). (**B**) A domain diagram illustrating the structural features of CDTa and their proposed roles. Sec indicates the proposed secretion signal; NUR the N-terminal unstructured region; pADPRT, the pseudo-ADP-ribosyltransferase domain; and ADPRT, the ADP-ribosyltransferase domain.

Structural and functional characterization of CDT, supplemented by studies conducted on the Iota toxin, has provided a much-needed basis for understanding how CDTb delivers CDTa into host cells. Initial studies on the holotoxin have revealed a binding mode that is different from distantly related toxins and have suggested the mechanism underlying toxin delivery is different (18, 24). These studies have also shed light on the dynamics of the CDTb SS-Loop and highlighted its potential role in delivering CDTa into host cells, although a functional understanding of the consequences of these interactions is required to fully appreciate its contribution (17). In this study, we provide additional structural and functional evidence to support the hypothesis that the CDTb SS-Loop plays a role during CDTa translocation. Central to this assertion was the identification of the KDKEK motif, which we have shown in close proximity to the CDTb SS-Loop. Notably, the interface between the CDTb SS-Loop and the KDKEK motif described here is small (we observe a single interaction formed between Asn491 of CDTb and Asp62 of CDTa) and unlikely to contribute to holotoxin assembly. Instead, we propose that the interaction between Asp62 of CDTa and Asn491 of CDTb directs the N-terminus of CDTa by introducing a “kink” within the N-terminus, leading to additional interactions between the CDTa N-terminal unstructured region and the SS-Loop of other CDTb protomers (Fig. 4). In support of this model, we have shown that mutating Asp62 and Glu64 does not change the dynamics of holotoxin assembly but does significantly impact the function of CDT. Introducing a kink into the N-terminus of CDTa also results in the positioning of residues 50–54 near the SS-Loop of CDTb_G_. Although the functional consequence of this interaction is, at present, not clear, we note that constructs of CDTa containing residues 50–54 exhibit greatly increased potency, an observation that underscores the potential role of the CDTb SS-Loop during intoxication. The benefit of restricting the CDTa N-terminus to interactions with the CDTb SS-Loop is not obvious from these data, and we note the CDTa N-terminus has been observed in other conformations, thus flexibility and dynamics likely govern the function of the SS-Loop (17). To that effect, additional studies are needed to fully interrogate how flexibility might be linked to its activity.

The mechanism of CDTa priming presented here takes advantage of two features, the KDKEK motif and residues 50–54 of the CDTa N-terminal unstructured region. Due to the high conservation of the KDKEK motif among Iota toxins, we expect this feature to play a key role in facilitating the activity of all three Iota family toxins (Fig. S5A). It is not clear if this motif correlates with features found in other binary toxins. For instance, because the enzymatic cargo of anthrax toxin is structurally and functionally distinct from the Iota toxins, the sequence of the N-terminal unstructured region is divergent. However, it should be noted that previous work on anthrax toxin led to the identification of two charged regions situated within the N-terminus of the anthrax toxin enzymatic components that are required for function. In the referenced work, it was shown that mutating these charged regions hindered translocation, a result that is attributed to charge-specificity within the anthrax toxin pore (30). However, a second application for the charged regions of anthrax toxin related to the role of the KDKEK motif cannot be immediately ruled out. Similarly, several studies conducted on anthrax toxin have established that a proton gradient maintained across the endosomal membrane is required for efficient toxin translocation (30–33). We would like to note that the data presented here do not rule out the possibility of a similar mechanism in CDT. Indeed, future studies conducted on CDT may provide insight into how the structural features we have identified fit into this paradigm.

In contrast to the KDKEK motif, only two of the residues that comprised the 50–54 segment of CDTa are conserved in other Iota family toxins (Fig. S5A), leading to additional questions about the role and specificity of this feature across species. Finally, the proposal that the SS-Loop plays a functional role is supported by its high sequence conservation across the family of Iota toxins. Indeed, a similar structure is observed within anthrax toxin, which has been found to interact with its cognate enzymatic cargo (34). Although the sequences across the structures are markedly different, we note that the presence of asparagine within these loops is invariable (Fig. S5D). To that end, we predict that interactions between toxin cargo and asparagine residues contained within these loops are central to the activity of binary toxins.

In summary, we present structural and functional data supporting the role of the CDTb SS-Loop in directing intoxication through interactions with two critical features within the CDTa N-terminus. The first, a sequence we describe as the KDKEK motif, was found to be vital for toxin function, and the second, a region formed by residues 50–59, has been shown to greatly enhance the potency of the toxin (Fig. 4B). Future studies are now needed to define how these segments participate during intoxication.

## MATERIALS AND METHODS

### Generation of CDTa constructs for purification

The gene encoding CDTa (*cdtA*) was amplified from *C. difficile R20291* genomic DNA by PCR, which was used to generate all constructs associated with this study. PCR products of each construct were inserted into the pGEX-6P1 vector using Gibson Assembly to generate expression vectors for CDTa_40-463_ (pSLMn0002), CDTa_50-463_ (pSLMn0003), CDTa_60-463_ (pSLMn0004), CDTa_67-463_ (pSLMn0008), CDTa_82-463_ (pSLMn0009), CDTa_95-463_ (pSLMn0010), CDTa_111-463_ (pSLMn0011), CDTa_118-463_ (pSLMn0012), and CDTa_134-463_ (pSLMn013). Mutants within the CDTa N-terminal unstructured region were encoded during PCR amplification and inserted into pGEX-6P1 using Gibson Assembly to yield the following vectors suitable for protein expression and purification: CDTa_Acidic_ (pSLMn007), CDTa_Basic_ (pSLMn006), CDTa_Hydrophobic_ (pSLMn005), CDTa_5XAla45-463_ (pSLMn0017), CDTa_5Ala55-463_ (pSLMn0018), CDTa_ADAAA_ (pSLMn0029), and CDTa_AAAEA_ (pSLMn0030).

### Purification of CDTa, CDTb, and CDTb oligomers

The construct encoding CDTa_40-463_ (pSLMn0002) was transformed into BL21 Gold pLysS *Escherichia coli* and was expressed as an N-terminal glutathione S-transferase (GST) fusion. The resulting strain was cultured at 37℃ until an optical density at 600 nm of 0.6–0.8 was reached, at which point protein expression was induced through the addition of 500 µM of isopropyl ß-D-1-thiogalactopyranoside (IPTG). The cultures were then incubated at 18℃ for 16–18 hours before harvesting by centrifugation. The resulting cell pellet was resuspended in 20 mM HEPES (pH 8.0) and 100 mM NaCl, passed through an Emulsiflex C3 to lyse, and the lysate was clarified by high-speed centrifugation. The supernatant was applied to glutathione agarose resin and washed twice with five column volumes resuspension buffer. CDTa was then eluted with five column volumes resuspension buffer supplemented with 10 mM glutathione. The eluate was dialyzed overnight at 4℃ to remove glutathione and simultaneously processed with H3C protease to remove the GST tag. The GST tag was recaptured with glutathione agarose resin, and the sample was further purified by size exclusion chromatography. All other CDTa constructs were purified following a virtually identical protocol.

A plasmid encoding residues 45–876 of CDTb (pBL870) was obtained from the laboratory of Dr. D. Borden Lacy (Vanderbilt University Medical Center) and was transformed into the BL21 RIL strain of *E. coli*. CDTb was then purified following a protocol similar to that which has been previously reported (22). After purification, CDTb was processed with trypsin (at a m/m ration of either 2.5:1 or 1.25:1, trypsin:CDTb) at 37℃ for 1 hour to activate the toxin. The trypsinization reaction was quenched with 0.1% phenylmethylsulfonyl fluoride (PMSF), and CDTb monomers and oligomers were isolated by size exclusion chromatography.

### ADP-ribosyltransferase activity assays

The ADP-ribosyltransferase activity of each construct used in this study was determined in a cellular milieu to mimic the environment inside the host cell. To prepare the cellular milieu, Caco-2 cells were grown at 37°C and 5% CO_2_ to ~90% confluency. The cells were washed twice with 1× phosphate buffered saline and resuspended in 50 mM HEPES (pH 8.0) and 150 mM NaCl before being lysed by gavage. The crude cellular lysate was centrifuged at 16,000 × *g* for 5 minutes to remove insoluble debris. The total protein concentration of the soluble fraction was determined by Coomassie assay. A total of 50 µg of protein was mixed with CDTa at a final concentration of 1 nM, and the mixture was allowed to incubate at 37°C for 8 hours. At the end of the incubation period, the reaction was quenched with Laemmli buffer and subsequently analyzed via immunoblot. ADP-ribose was detected using a rabbit anti-poly/mono-ADP-ribose primary antibody (Cell Signaling #89190) and goat anti-rabbit IRDye CW800 secondary antibody (Licorbio 926-32211). Simultaneously, the amount of GAPDH in each reaction was determined using a mouse anti-GAPDH primary antibody (Santa Cruz 6C5 sc-32233) and anti-mouse IRDye 680RD secondary antibody (Licorbio 926-68070) as a loading control.

### Cytotoxic cell-rounding assays

CDT cytotoxicity assays were performed using human colorectal carcinoma cells (Caco-2, ATCC HTB-37) grown in Eagle’s minimum essential media (EMEM), supplemented with 20% fetal bovine serum and maintained in an atmosphere of 5% CO_2_ at 37℃. Caco-2 cells were plated on transparent 96-well plates and grown to a confluency of ~90%. Before the addition of CDT, the media were removed, and the cells were washed with 1× PBS. For end point assays, CDT was added to each well to achieve final concentrations of 8.6 nM CDTb and 100 nM CDTa. The 96-well plate was then transferred into a Tecan Spark Cyto Multimode Plate Reader maintained at 37°C with 5% CO_2_. Images were then collected for each condition. To determine the efficiency of each construct, a nearly identical experimental setup was used with modifications to the final concentration of CDTa in each well, and images were taken every 20 minutes over a period of 5 hours. All data were quantified using the Weka Segmentation program in FIJI (35, 36). Briefly, a model was generated to enumerate the number of flat and round cells in each image. The number of each cell type was automatically counted in FIJI using the “Analyze Particles” plugin to determine the ratio of round cells:flat cells (35).

### Biolayer interferometry

CDTb oligomers were purified as described above and functionalized with N-hydroxy succinimide biotin at a 4:1 stoichiometry for 2 hours at room temperature. The reaction was quenched by the addition of 100 mM Tris pH 8.0, and the free biotin was removed via size exclusion chromatography. Streptavidin biosensors were then saturated with biotinylated CDTb oligomers using a Sartorious Octet Red 96e. The saturated biosensors were then washed with binding buffer (20 mM HEPES, pH 8.0, 100 mM NaCl) and were blocked with binding buffer supplemented with 0.01% Igepal CA-630 and 0.1% bovine serum albumin. The biosensors were then immersed in solutions containing CDTa ranging in concentration from 200 to 0.032 nM. CDTa was allowed to associate with CDTb for 5 minutes before the biosensor was removed from the CDTa-containing solution and immersed in binding buffer for 10 minutes to monitor dissociation. The resulting data were then fit to an exponential growth model for the association step to derive equilibrium rate constants (k_obs_) for all concentrations assayed. The k_obs_ was plotted against the concentration of CDTa in each solution, and the data were fit to a line. The rate of association was derived from the slope of the fit line (37). The dissociation constant was derived for each construct at equilibrium. In brief, the signal obtained for each construct at equilibrium was averaged and plotted against concentration. The data were then fit to a sigmoidal dose-response model to derive the K_D_.

### Cryo-EM sample preparation, data processing, and analysis

To generate CDT, CDTa_50-463_ and CDTb were mixed at a ratio of 1:7 (CDTa:CDTb) and were prepared at a final concentration of 1 µM holotoxin. CDT was then applied to Quantifoil R2/1 200 mesh copper grids and flash frozen in liquid ethane using a Vitrobot Mark IV. A total of 14,464 movies were collected on a Titan Krios equipped with a Gatan K3 direct electron detector and GIF energy filter set to a slit width of 20 eV using EPU (Thermo Fisher). All data were collected at a pixel size of 0.65 Å/pixel, a dose of 52 e^-^/Å^2^, and defocus values ranging from −0.5 to −2.5 µm. The movies were aligned with MotionCorr2, and the defocus values were estimated using CTFFIND4 (38, 39). All subsequent processing was conducted in CryoSPARC (40). To generate a three-dimensional volume, a total of 3,881 particles were first picked by hand and used to generate templates to autopick the entire data set in CryoSPARC. The resulting particle stack was refined through two rounds of 2D classification followed by a single round of 3D homogenous refinement. A mask of CDTa was constructed from this initial volume and applied to three additional rounds of classification. The final map was generated using a non-uniform refinement (41). A model of the interaction between CDTa_50-463_ and CDTb was constructed using the structure of CDTa_60-463_ in complex with CDTb as a template (PDB 6V1S) (18). The model was iteratively built by hand in Coot v 0.9.8.1, refined, and validated in Phenix v 1.20.1 (42–46).

## Data Availability

The structural data reported here have been made available through the Electron Microscopy Data Bank (EMDB-44419) and Protein Data Bank (PDB 9BBF).

## SUPPLEMENTAL MATERIAL

Supplemental Material mbio.02405-24-s0001.pdf Supplemental figures and table.

ASM does not own the copyrights to Supplemental Material that may be linked to, or accessed through, an article. The authors have granted ASM a non-exclusive, world-wide license to publish the Supplemental Material files. Please contact the corresponding author directly for reuse.
