# Supplementary material for: The N-terminus of the Clostridioides difficile transferase A component directs toxin activity and potency
Source: mBio. 2024 Nov 29;16(1):e02405-24. doi: 10.1128/mbio.02405-24 (PMC11708034; doi:10.1128/mbio.02405-24)
Supplement: Supplemental Material — Supplemental figures and table. [file mbio.02405-24-s0001.pdf]

SUPPLEMENTAL DATA

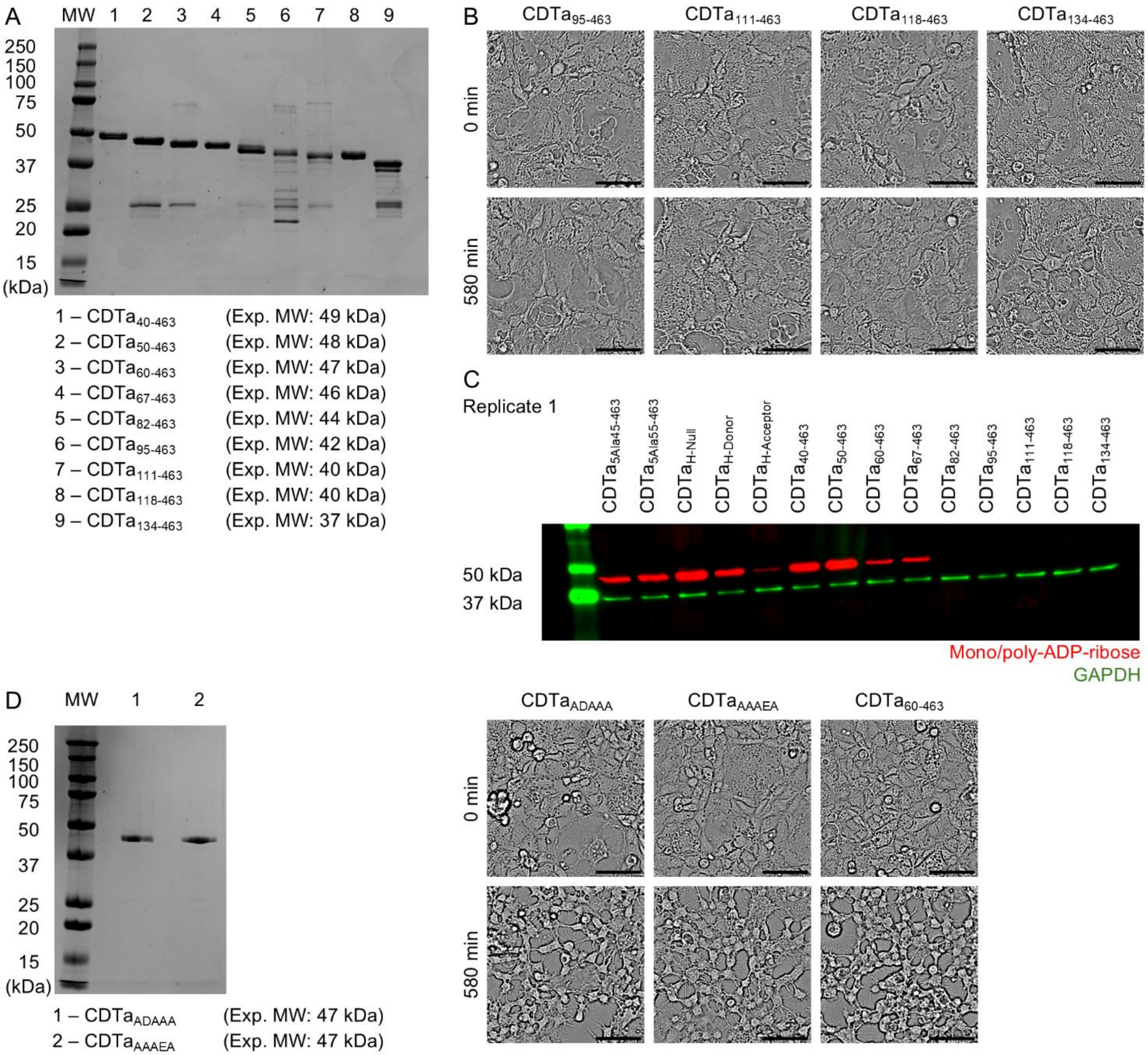

Supplemental Fig. 1 – **Purification and Characterization of Various CDTa Constructs**. (**A**) An SDS-PAGE gel showing the relative purity of nine of the CDTa constructs assayed. (**B**) Intoxication of Caco-2 cells with CDTa$_{94-463}$, CDTa$_{111-463}$, CDTa$_{118-463}$, and CDTa$_{134-463}$. None of these constructs were found to be active over 580-minutes. The scale bar indicates a distance of 100 µm. (**C**) ADP-ribosylation activity of each CDTa construct used in this study. The signal in the red channel corresponds to reactivity with an anti-mono/poly-ADP-ribose antibody and the green channel corresponds to reactivity with an anti-GAPDH antibody. (**D**) An SDS-PAGE gel showing the relative purity of CDTa$_{ADAAA}$ and CDTa$_{AAAEA}$ (left). Cytopathic cell rounding of the CDTa$_{ADAAA}$ and CDTa$_{AAAEA}$ constructs on Caco-2 cells compared to CDTa$_{60-463}$ (right).

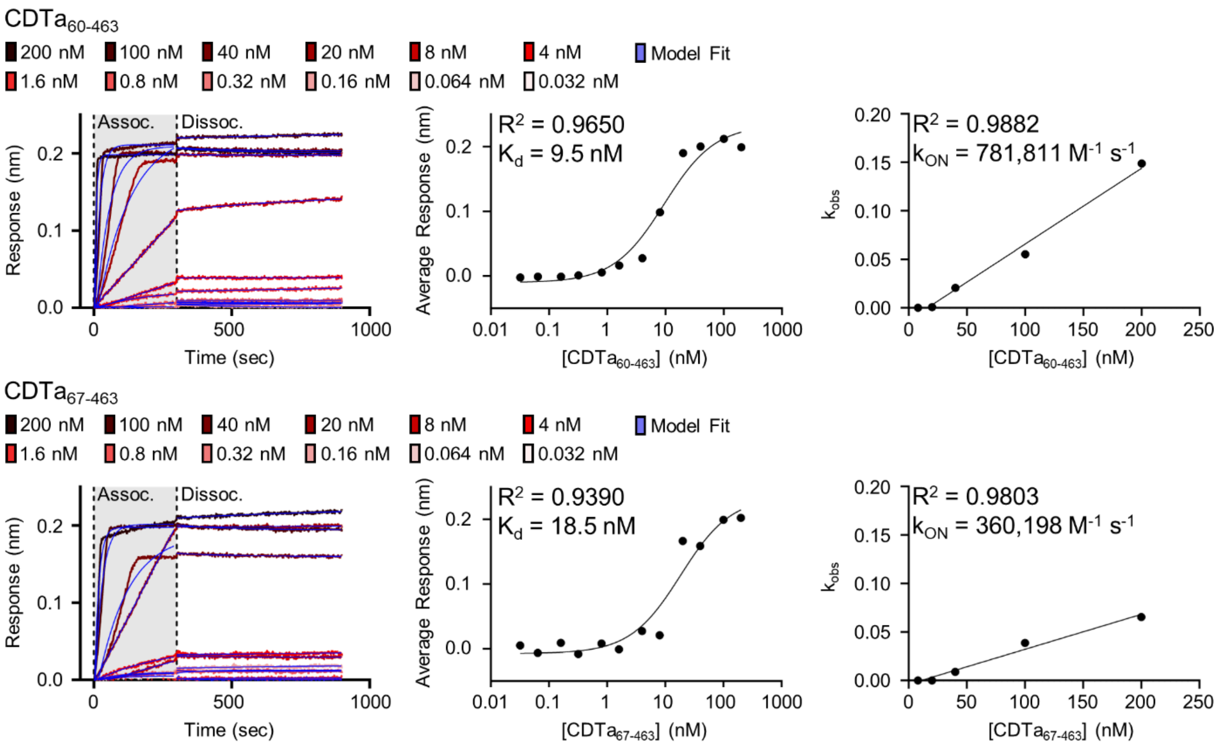

Construct	K_d (nM) [CI95]	Change	k_{ON} (M^{-1} s^{-1}) [CI95]	Change*
$CDTa_{60\text{-}463}$	9.5 [5.4 to 17.0]	-	8×10^5 [8×10^5 to 9×10^5]	-
$CDTa_{67\text{-}463}$	18.5 [8.4 to 42.3]	1.9	4×10^5 [2×10^5 to 5×10^5]	2

Supplemental Fig. 2 – Affinity of Complex Maturation $CDTa_{60\text{-}463}$ and $CDTa_{67\text{-}463}$. The affinity of each construct for oligomeric CDTb was assessed using biolayer interferometry. The concentration of CDTa used is indicated in red for each construct. All data were fit to an exponential growth curve for the association phase (indicated in the gray background) and an exponential decay model for the dissociation phase. The affinity of each CDTa construct was determined at equilibrium. For each construct, the observed response was fit to a dose-response model to derive the dissociation constant (K_d, center panel). The apparent rate of association was derived for each concentration tested (k_{obs}). The k_{obs} was plotted against concentration to derive the rate of association (k_{ON}, right panel). The calculated dissociation constants (Kd) and rates of association (k_{ON}) for each construct are indicated in the table below. Reported to the right of each value is the change in each value with respect to $CDTa_{60\text{-}463}$.

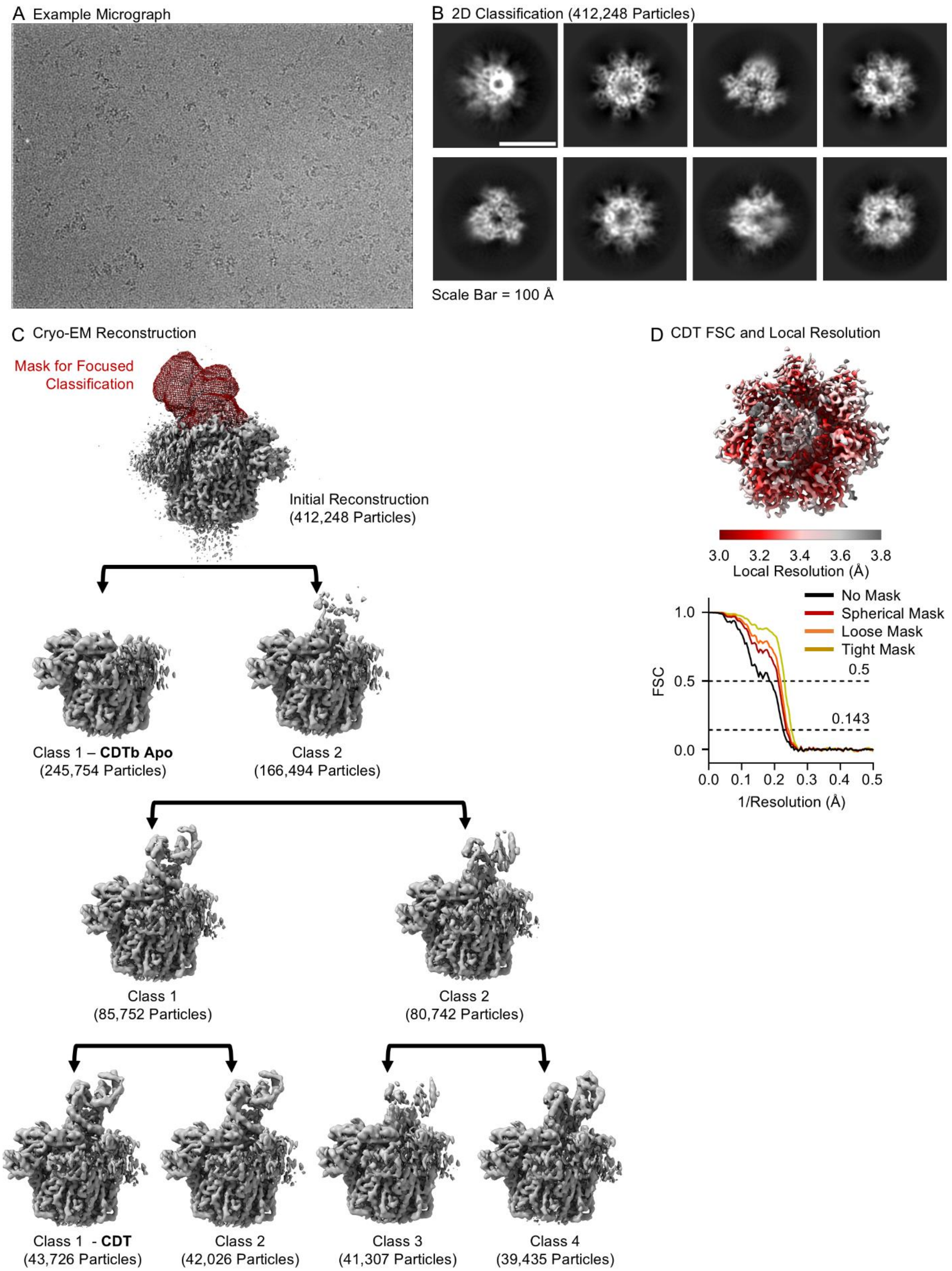

Supplemental Fig. 3 – Cryogenic Electron Microscopy Analysis of Oligomeric CDTb in Complex with CDTa$_{50\text{-}463}$.

(**A**) A representative micrograph of CDTa$_{50\text{-}463}$ in complex with oligomeric CDTb. (**B**) Representative 2D classes of the complex showing an array of orientations as observed in this dataset. (**C**) The ab initio map reconstructed from this

sample with a mask of CDTa shown in red mesh. This mask was used during all subsequent 3D classifications. The data were further processed over three rounds of focused 3D classification to select for structures bound to CDTa. (**D**) Maps were reconstructed of CDTb Apo (top) and CDTb bound to $CDTa_{50-463}$ (CDT, lower left). The local resolution estimates and corresponding FSC curve of the CDT map are indicated.

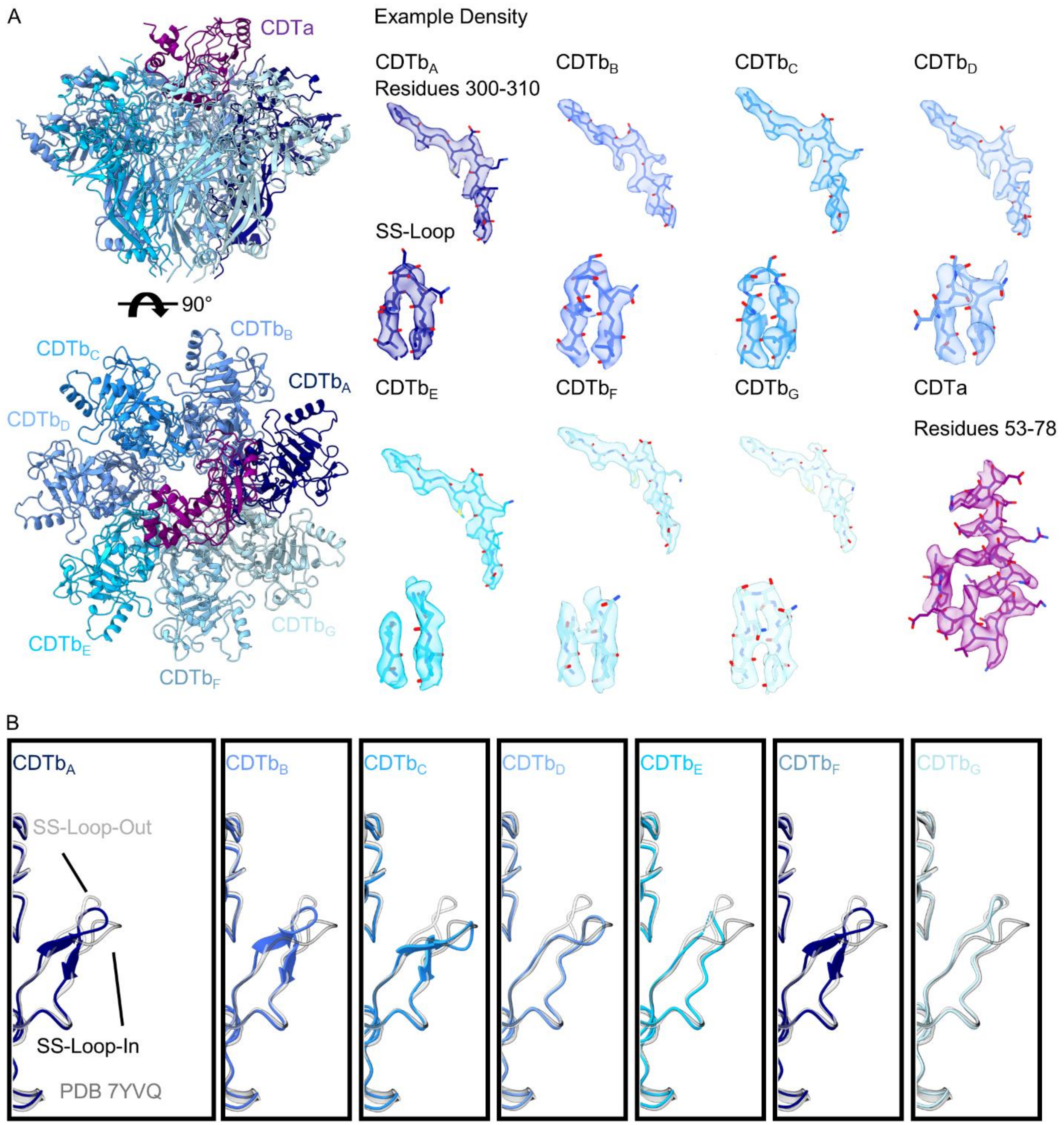

Supplemental Fig. 4 – (**A**) The model of $CDT_{50\text{-}463}$ constructed from the Cryo-EM density generated as a part of this study. CDTa is shown in purple and CDTb in various shades of blue. Density is shown for residues 300-310 and the SS-Loop of CDTb to demonstrate the goodness of fit for this model. Similarly, density corresponding to residues 53-78 of CDTa is shown in purple as an example. (**C**) The conformation of the SS-Loop of all seven chains of CDTb compared to the Loop-In and Loop-Out conformations previously determined (PDB 7YVQ, shown in gray).

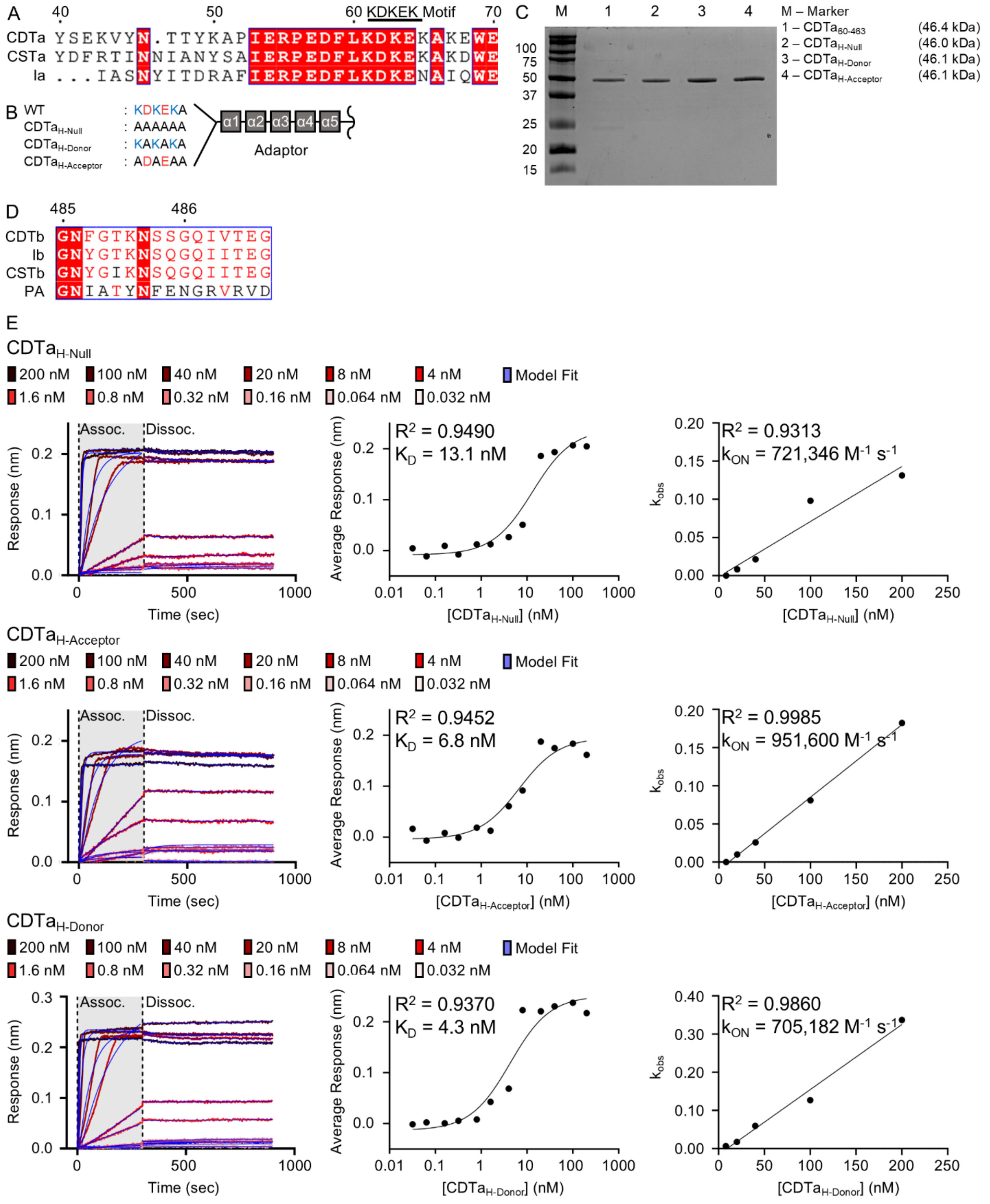

Construct	K_D (nM) [CI95]	Change	k_{ON} (M^{-1} s^{-1}) [CI95]	Change*
CDTa$_{H\text{-}Null}$	13.4 [6.5 to 26.8]	1.4	7 x 10^5 [4 x 10^5 to 11 x 10^5]	0.9
CDTa$_{H\text{-}Acceptor}$	6.8 [3.3 to 14.0]	0.7	10 x 10^5 [9 x 10^5 to 10 x 10^5]	1.3
CDTa$_{H\text{-}Donor}$	4.3 [2.0 to 9.3]	0.5	7 x 10^5 [13 x 10^5 to 21 x 10^5]	2.1

Supplemental Fig. 5 – Design, Purification, and Characterization of CDTa$_{H\text{-}Acceptor}$, CDTa$_{H\text{-}Donor}$, and CDTa$_{H\text{-}Null}$. (**A**) A sequence alignment of the N-terminus of CDTa (*C. difficile*), CSTa (*C. spiroforme*), and Ia (*C. perfringens*). Conserved residues are indicated in red. (**B**) A schematic illustrating the design of CDTa$_{H\text{-}Acceptor}$, CDTa$_{H\text{-}Donor}$, and CDTa$_{H\text{-}Null}$. WT

denotes the wild type sequence. (**C**) An SDS-PAGE gel demonstrating the purity of $CDTa_{H\text{-}Acceptor}$, $CDTa_{H\text{-}Donor}$, and $CDTa_{H\text{-}Null}$. (**D**) A sequence alignment of the SS-Loop within the Iota toxin family (CDTb, Ib, and CSTb) compared to the distantly related anthrax toxin protective antigen (PA). (**E**) The affinity of each construct for oligomeric CDTb was assessed using biolayer interferometry. The concentration of CDTa used is indicated in red for each construct assessed. All data were fit to an exponential growth curve for the association phase (indicated in the gray background) and an exponential decay curve for the dissociation phase. The affinity of each CDTa construct was determined at equilibrium. For each construct, the observed response was fit to a dose-response model to derive the dissociation constant (K_D, center panel). The apparent rate of association was derived for each concentration tested (k_{obs}). The k_{obs} was plotted against concentration to derive the rate of association (k_{ON}, right panel). The calculated dissociation constants (K_D) and rates of association for various CDTa constructs used throughout this study are indicated in the table below. Reported to the right of each value is the change in each value with respect to $CDTa_{60\text{-}463}$.

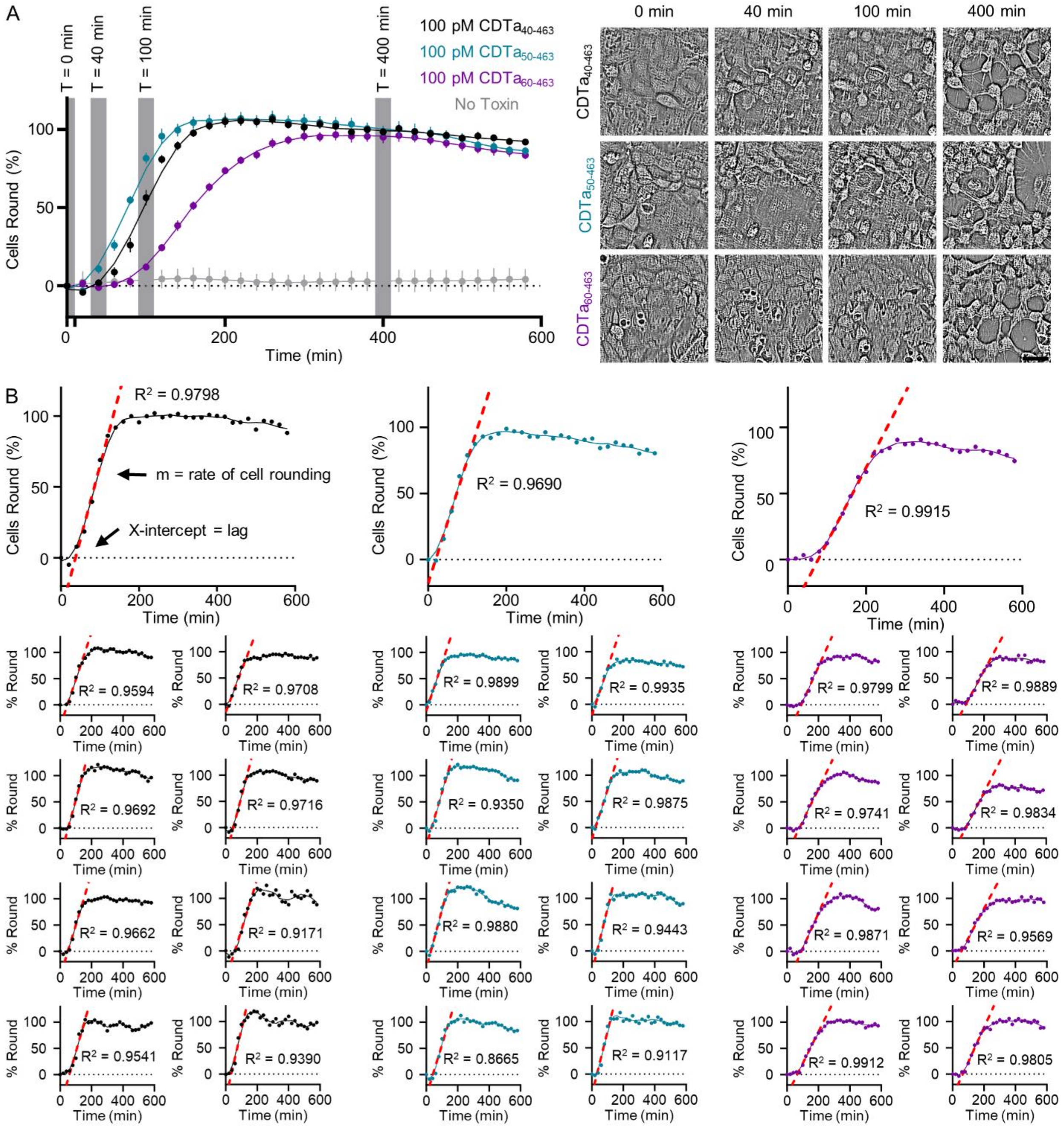

Supplemental Fig. 6 – **Activity of CDTa40-463, CDTa50-463, and CDTa60-463 at 100 pM**. (**A**) A comparison of $CDTa_{40\text{-}463}$ (black), $CDTa_{50\text{-}463}$ (cyan), and $CDTa_{60\text{-}463}$ (purple) at a final concentration of 100 pM (left). Error bars represent standard error in measurement. Representative images obtained during the assay for each construct obtained at 0 minutes, 40 minutes, 100 minutes, and 400 minutes for each construct assayed. (**B**) The activity of each construct was quantified as the percent of round cells per image. The percentage of round cells was plotted against time for each assay condition and data fit to a straight line. The rate of cell rounding was defined as the slope of the line and the lag time as the X-intercept. Each replicate was fit independently to generate a unique measurement.

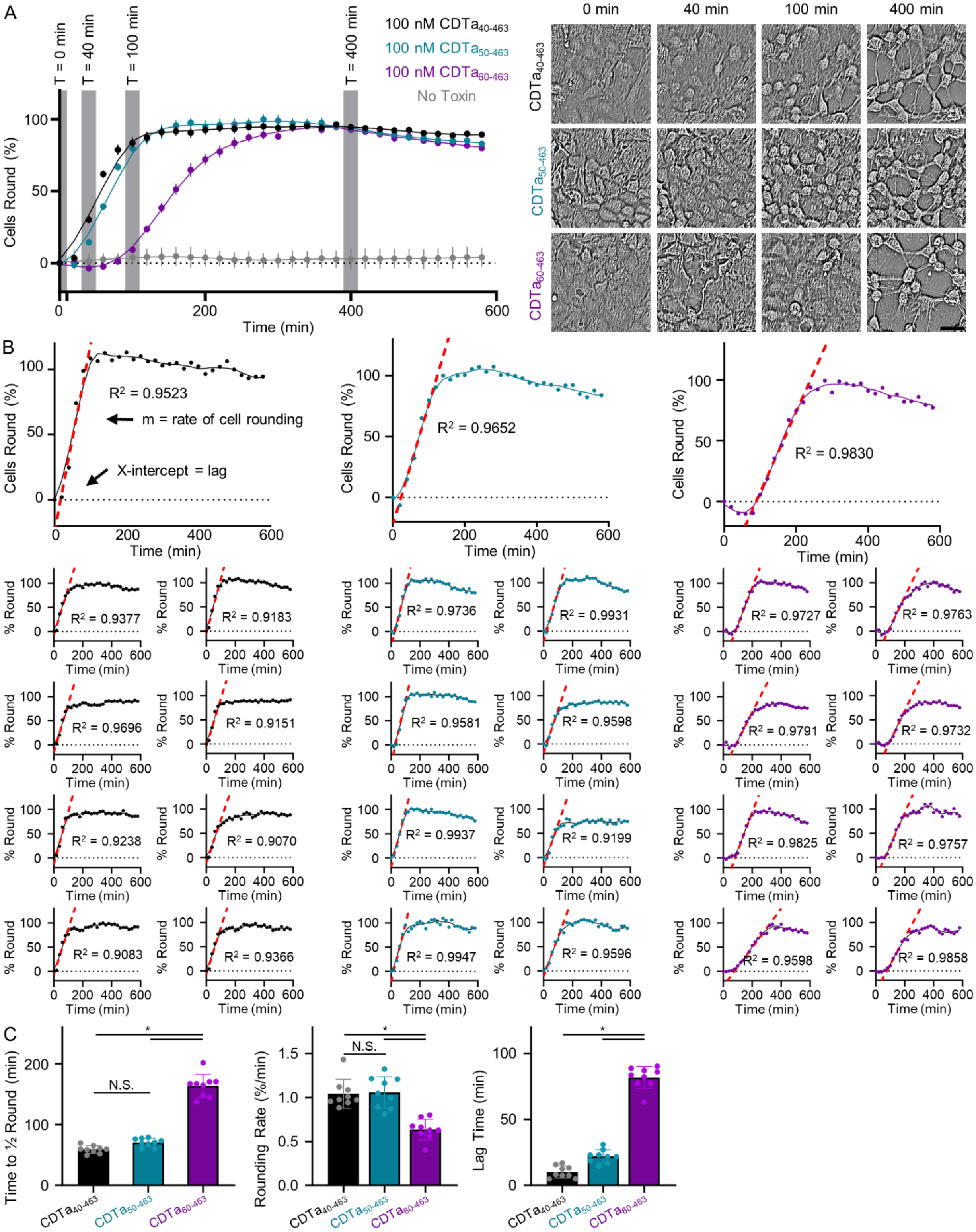

Construct	Time to 1/2 Round (min)	Change	Rounding Rate (%/min)	Change	Lag (min)	Change
CDTa$_{40\text{-}463}$	59 ± 6	-	1.0 ± 0.2	-	10 ± 5	-
CDTa$_{50\text{-}463}$	70 ± 7	1.2	1.1 ± 0.2	1.1	22 ± 5	2.2
CDTa$_{60\text{-}463}$	163 ± 19	2.8	0.6 ± 0.1	0.6	82 ± 8	8.2

Supplemental Fig. 7 – Activity of CDTa$_{40\text{-}463}$, CDTa$_{50\text{-}463}$, and CDTa$_{60\text{-}463}$ at 100 nM. (**A**) A comparison of CDTa$_{40\text{-}463}$ (black), CDTa$_{50\text{-}463}$ (cyan), and CDTa$_{60\text{-}463}$ (purple) at a final concentration of 100 nM (left). Error bars represent standard

error in measurement. Representative images obtained during the assay for each construct obtained at 0 minutes, 40 minutes, 100 minutes, and 400 minutes for each construct assayed. (**B**) The activity of each construct was quantified as the percent of round cells per image. The percentage of round cells was plotted against time for each assay condition and data fit to a straight line. The rate of cell rounding was defined as the slope of the line and the lag time as the X-intercept. Each replicate was fit independently to generate a unique measurement. (**C**) Quantified results for all three constructs assayed at 100 nM. Averages are included in the tables below each graph. The fold-change in comparison to $CDTa_{40-463}$ is shown to the right of each derived value.

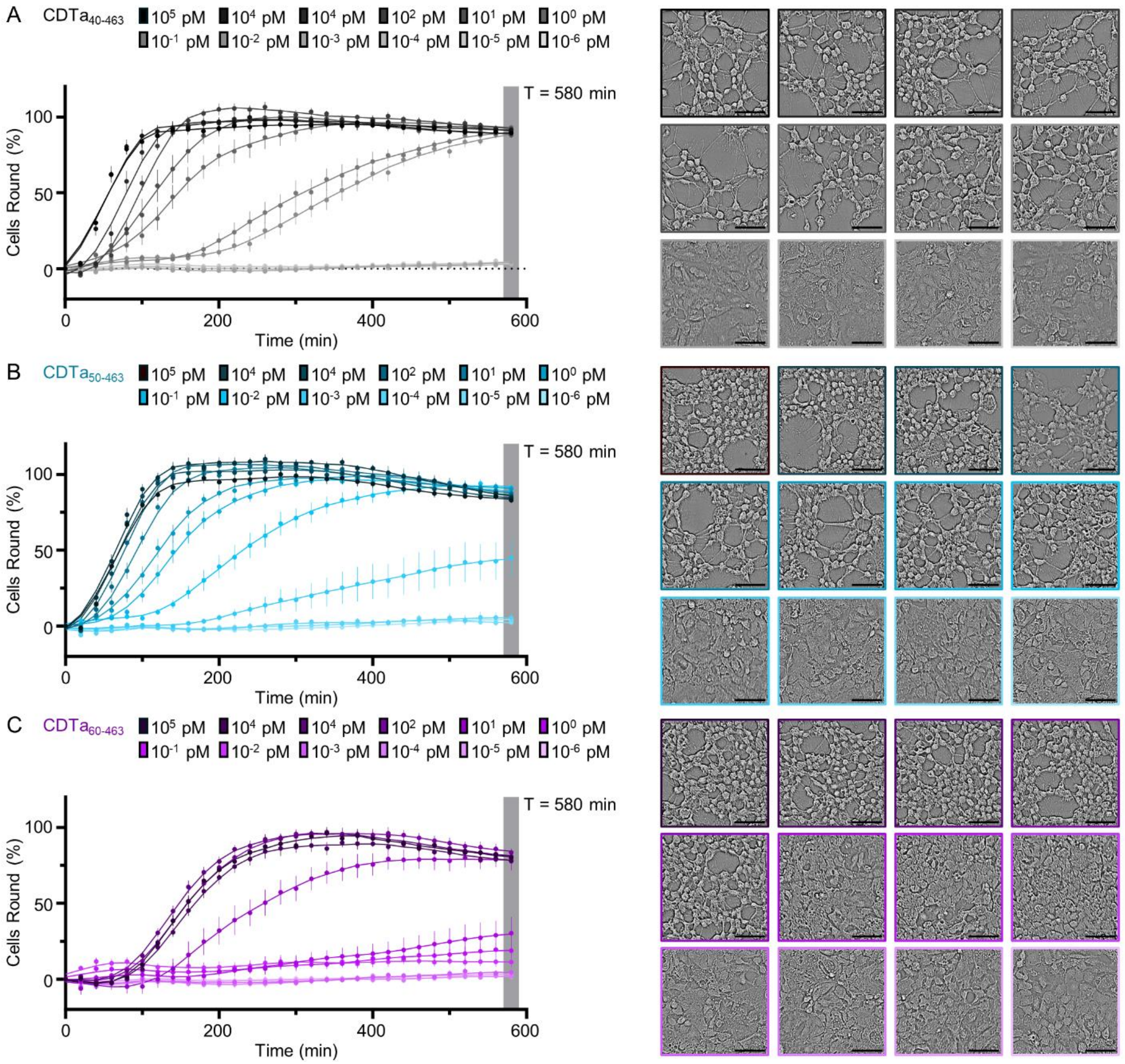

Supplemental Fig. 8 – **Determination of the Effective Concentration of CDTa$_{40\text{-}463}$, CDTa$_{50\text{-}463}$, and CDTa$_{60\text{-}463}$.** The activity of each construct was assayed at concentrations ranging from 100,000 pM to 0.000001 pM. Data were quantified as the percentage of cells in each image that had rounded in response to intoxication. Error bars represent standard error in measurement. Images collected at 580-minute for each concentration are shown to the right of each plot. The corresponding border indicates the concentration of CDTa used. Scale bars denote a distance of 100 µm.

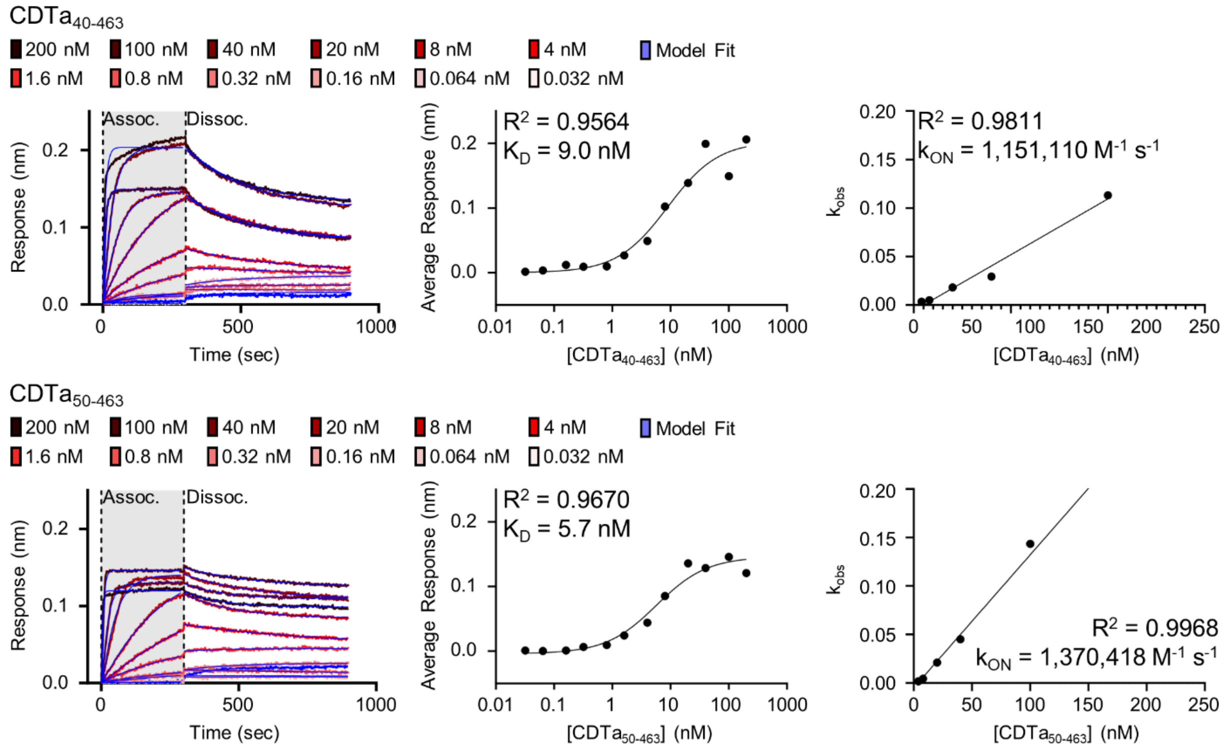

Construct	K_D (nM) [CI95]	Change	k_{ON} (M^{-1} s^{-1}) [CI95]	Change*
$CDTa_{40-463}$	9.0 [4.5 to 18.2]	0.9	12 x 10^5 [9 x 10^5 to 15 x 10^5]	1.5
$CDTa_{50-463}$	5.7 [3.2 to 10.2]	0.6	14 x 10^5 [1.3 x 10^6 to 14 x 10^5]	1.8

Supplemental Fig. 9 – Affinity of $CDTa_{60-463}$ and $CDTa_{67-463}$ for Oligomeric CDTb. The affinity of each construct for oligomeric CDTb was assessed using biolayer interferometry. The concentration of CDTa used is indicated in red for each construct. All data were fit to an exponential growth curve for the association phase (indicated in the gray background) and an exponential decay model for the dissociation phase. The affinity of each CDTa construct was determined at equilibrium. For each construct, the observed response was fit to a dose-response model to derive the dissociation constant (K_d, center panel). The apparent rate of association was derived for each concentration tested (k_{obs}). The k_{obs} values were plotted against concentration to derive the rate of association (k_{ON}, right panel). The calculated dissociation constants (Kd) and rates of association (k_{ON}) for each construct are indicated in the table below. Reported to the right of each value is the change in each value with respect to $CDTa_{60-463}$.

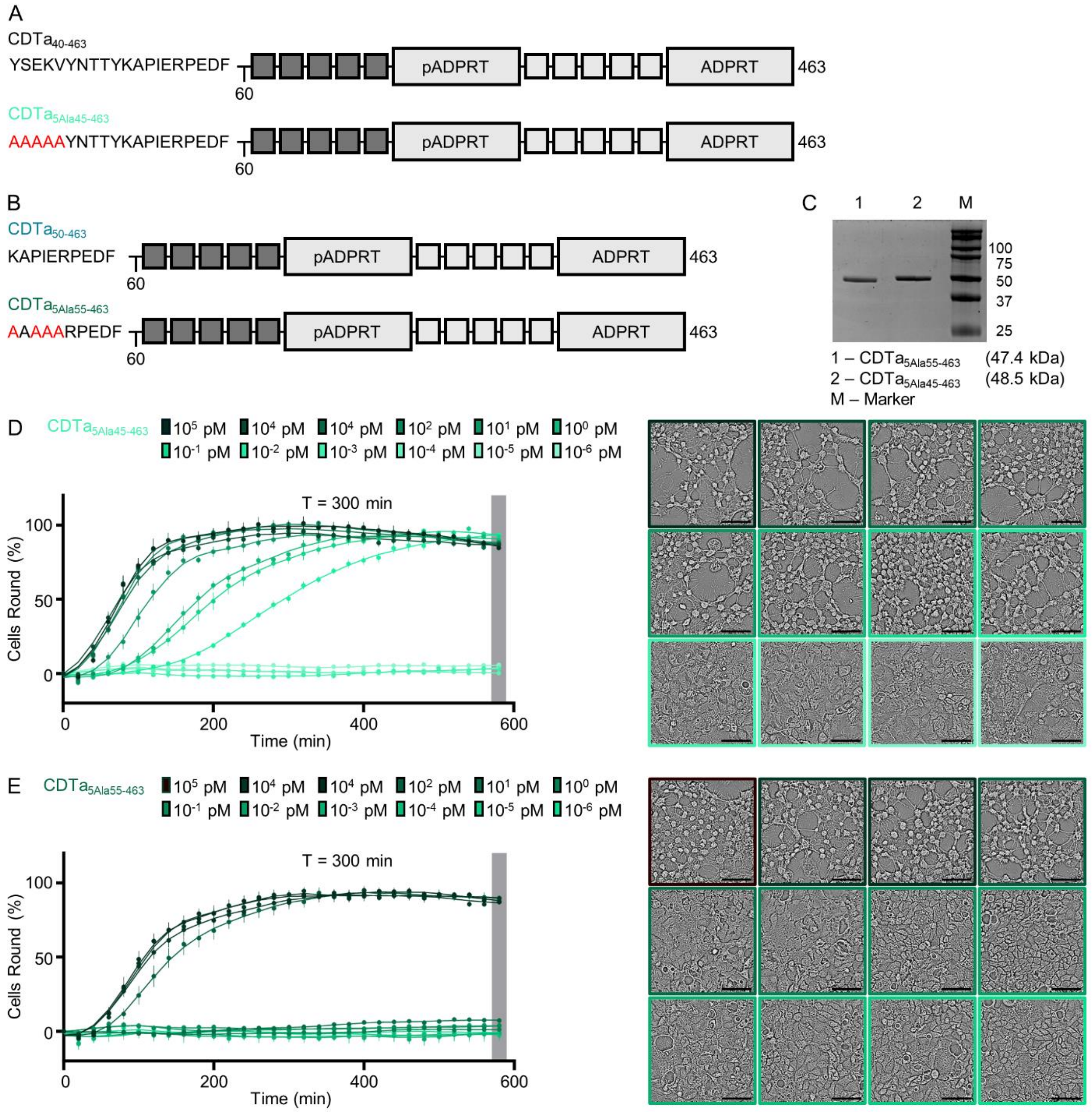

Supplemental Fig. 10 – Determination of the Effective Concentration of CDTa$_{5Ala45-463}$ and CDTa$_{5Ala55-463}$. A schematic illustrating the design of the CDTa$_{5Ala45-463}$ compared to CDTa$_{40-463}$ (**A**) and CDTa$_{5Ala55-463}$ compared to CDTa$_{50-463}$ (**B**). (**C**) An SDS-PAGE gel indicating the relative purity of CDTa$_{5Ala45-463}$ and CDTa$_{5Ala45-463}$. (**D**) The activity of CDTa$_{5Ala45-463}$ at concentrations ranging from 100,000 pM to 0.000001 pM. Error bars represent standard error in measurement. Representative images are shown for each concentration on the right. Scale bar denotes a distance of 100 µm. (**E**) The activity of CDTa$_{5Ala45-463}$ at concentrations ranging from 100,000 pM to 0.000001 pM. Error bars represent standard error in measurement. Scale bar denotes 100 µm. Representative images are shown for each concentration on the right.

Supplemental Table 1 – Cryo-EM Data Processing and Model Statistics

Data Collection and Processing

	**CDTa$_{50\text{-}463}$ + CDTb**
EMDB	44419
Voltage (kV)	300
Electron exposure (e-/Å2)	52
Defocus range	-0.5 to -2.5
Pixel size (Å)	0.65
Symmetry imposed	C1
Initial particle images (no.)	412,248
Final particle images (no.)	43,726
Map resolution (Å)	3.6
FSC Threshold	0.143
**Refinement**	
PDB	9BBF
Initial model used (PDB)	6V1S
Model resolution (Å)	3.6
FSC threshold	0.143
Map sharpening *B* factor (Å2)	129.5
Model composition	
Non-hydrogen atoms	19,880
Protein residues	2,533
Ligands	14
B factors (Å2)	
Protein residues	77.78
Ligands	78.36
R.m.s deviations	
Bond lengths (Å)	0.004
Bond angles (°)	0.705
Validation	
MolProbity score	1.88
Clashscore	9.08
Poor rotamers (%)	0.45
EMRinger Score	2.25
Ramachandran plot	
Favored (%)	94.2
Allowed (%)	5.8
Disallowed (%)	0.0
Ramachandran Z-score	
Whole	-1.44
Helix	-1.00
Sheet	-0.18
Loop	-1.29
